# Crying in Children

**Published:** 1893-11

**Authors:** 


CRYING IN CHILDREN.—The cry of children, according to Dr. E. C. Hill, in pneumonia and capillary bronchitis is moderate and peevish and muffled as if the door were shut between child and hearer. The cry of croup is hoarse, brassy, and metallic, with a crowing inspiration. That of cerebral disease, particularly hydrocephalus, is short, sharp, shrill, and solitary. Marasmus and tubercular peritonitis are manifested by moaning and wailing. Obstinate, passionate, and long-continued crying tells of earache, thirst, hunger, original meanness, or the pricking of a pin. The pleuritic is louder and shriller than the pneumonic, and is evoked by moving the child, or on coughing. The cry of intestinal ailments is often accompanied by wriggling and writhing before defecation. Exhaustion is manifested with a whine. Crying only, or just after coughing, indicates pain caused by the act. The return or inspiratory part of the cry grows weaker toward the fatal end of all disease, and the absence of crying during disease is often of graver import than its presence, showing complete exhaustion and loss of power. Loud screaming sometimes tells of renal gravel.—*Ontario Medical Journal.*

